# Evaluation of factors predicting tinnitus outcomes following cochlear implantation: Protocol for a prospective quasi-experimental study

**DOI:** 10.1371/journal.pone.0302790

**Published:** 2024-06-17

**Authors:** Bas Labree, Magdalena Sereda, Helen Cullington, Susan Johnson, Paige Church, Josephine Dunster, Derek J. Hoare

**Affiliations:** 1 NIHR Nottingham Biomedical Research Centre, Nottingham, United Kingdom; 2 Hearing Sciences, Mental Health and Clinical Neurosciences, School of Medicine, University of Nottingham, Nottingham, United Kingdom; 3 Auditory Implant Service, University of Southampton, Southampton, United Kingdom; 4 Nottingham Auditory Implant Programme, Nottingham University Hospitals NHS Trust, Nottingham, United Kingdom; 5 NIHR Clinical Research Network (CRN) East Midlands, Nottingham Health Science Partners, Nottingham, United Kingdom; 6 Independent Researcher, Nottingham, United Kingdom; University of Glasgow, UNITED KINGDOM

## Abstract

Cochlear implantation is an effective intervention to restore useful aspects of hearing function in adults with severe-to-profound hearing loss. Tinnitus, the perception of sound in the absence of an external source, is common in people with severe-to-profound hearing loss. Existing evidence suggests cochlear implantation may be effective in reducing the negative impact of tinnitus in this population. However, this is contradicted by data suggesting that up to half of cochlear implant recipients experience tinnitus, and that some of these patients who did not have tinnitus before cochlear implantation experience it after surgery or cochlear implant activation. Most evidence on the effects of cochlear implantation on tinnitus comes from secondary data in cochlear implant studies primarily concerned with hearing-related outcomes. Hence, the quality of the evidence for effects on tinnitus is low and not suitable to inform clinical recommendations or decision-making. This study will systematically collect data on tinnitus and tinnitus-related outcomes from patients at multiple points during the cochlear implant pathway to characterise changes in tinnitus. This will improve our understanding of the effects of cochlear implantation for tinnitus in adults with severe to profound hearing loss and inform the design of clinical trials of cochlear implantation for tinnitus.

## Introduction

Unilateral cochlear implantation is a clinically- and cost-effective intervention to restore useful aspects of hearing function in adults with severe-to-profound hearing loss in both ears [1]. Cochlear implant recipients generally have an awareness of environmental sounds [2], and a good understanding of conversational speech in quiet listening conditions [3]. In addition to hearing loss, adults eligible to receive a cochlear implant often experience tinnitus: a perception of sound in the ears or head that does not come from an external source [4]. Tinnitus is associated with psychological symptoms such as anxiety, depression, and insomnia, which can have a significant impact on the patient’s quality of life [5, 6]. Population-based studies suggest a prevalence of tinnitus among cochlear implantation candidates to be at least 50%, but it may be as high as 80% [7, 8]. Systematic reviews suggest that in addition to its effects on hearing-related outcomes, cochlear implantation can alleviate tinnitus [9–15]. However, previous data from systematic reviews and population-based studies also suggests that roughly half of cochlear implant recipients still experience tinnitus after implantation [8, 16, 17]. Moreover, a small proportion of patients have been found to develop or experience a worsening of their existing tinnitus after implantation [14]. In the UK, cochlear implantation is not currently recommended as a primary intervention for tinnitus [18].

The availability of high-quality evidence from large-scale, multicentre trials of cochlear implantation for tinnitus is lacking. For instance, National Institute for Health and Care Excellence (NICE) guidelines and systematic reviews identified the lack of randomised controlled trials and high-quality prospective studies on the effectiveness of cochlear implantation for tinnitus in people with bilateral, severe-to-profound hearing loss. In the evidence on the effects of cochlear implantation on tinnitus that is available, there is a large degree of heterogeneity [9, 13, 18]. This is driven by two factors. The first is variability in the data collection methods and the types of outcome measures used to assess tinnitus and related characteristics. Whilst current efforts in the wider field of tinnitus research focus on improved systematic data collection using standard outcome measures [19, 20], outcome measures used in studies looking at the effects of cochlear implantation for tinnitus are not standardised. Prospective, systematic, and comprehensive collection of tinnitus outcomes is not routinely undertaken in patients undergoing cochlear implantation. Instead, tinnitus is typically assessed as a secondary measure in clinical trials that are primarily concerned with cochlear implantation as an intervention to restore useful aspects of hearing. These trials tend not to collect data about comorbid outcomes such as anxiety, depression, or insomnia. As a result, it is often not possible to fully characterise changes in tinnitus or dissociate them from changes in hearing-related problems following implantation from the available evidence. The second factor is the evidence gap that arises from specific challenges regarding the feasibility of conducting clinical trials of cochlear implantation for the alleviation of tinnitus. It is not possible to fully randomise patients because implantation cannot be withheld from patients who are eligible to receive a cochlear implant based on the current candidacy criteria. Randomising patients in an RCT to receive either one or two CIs has been done [21], but such a design lacks a true control condition since participants in both groups are undergoing cochlear implantation. An alternative approach using a waiting list control design could be unethical due to the potential negative effect of delaying cochlear implantation on patient outcomes [22]. Furthermore, the nature of cochlear implantation makes it impossible to blind patients and clinicians to this intervention.

The lack of consistent and comprehensive data also prevents the disentanglement of changes in tinnitus following implantation from the beneficial effects of implantation on hearing and quality of life [23]. Crucially, it is a barrier to predicting tinnitus outcomes following cochlear implantation, and thus cannot support evidence-based decision making by patients and clinicians or lead to changes in practice for the management of tinnitus in patients with bilateral severe-to-profound hearing loss [10, 11, 13, 14]. Whilst findings from small observational studies suggest a potential association between surgical, or pre- and post-operative psychological symptoms during auditory rehabilitation and the occurrence or worsening of tinnitus and related symptoms, the relevant factors, or the stages of cochlear implantation care pathway at which these factors might influence tinnitus, remain unknown [24, 25]. Consequently, it is unclear whether these factors require management during the cochlear implantation pathway. Taken together, there is a need for systematic and comprehensive research to address the current evidence gap and to improve our understanding of meaningful changes in tinnitus and related characteristics in patients undergoing cochlear implantation.

The purpose of the present study is to determine what changes in tinnitus and related outcomes occur in patients following cochlear implantation. Systematic data collection at different time points during the implantation care pathway will provide estimates of prevalence, incidence of new and worsening tinnitus and access to tinnitus care, and data on the factors associated with changes in tinnitus-related outcomes. This will allow exploration of the utility of different outcomes as predictors of tinnitus changes and tinnitus-related cochlear implantation candidacy criteria in future studies. Moreover, the data will be used to explore associations between tinnitus and factors related to hearing, cochlear implantation, psychological wellbeing, and quality of life in cochlear implant recipients with and without tinnitus [23].

## Methods

This study received favourable opinion from the Bromley Research Ethics Committee (REC) Health Research Authority (HRA) and Health and Care Research Wales (HCRW) on 26/04/2023 (amended 18/06/2023) IRAS ID: 292855, protocol number: 23004, REC reference: 23/LO/0140. This study is sponsored by the University of Nottingham, sponsor reference: 23004. This protocol is registered on clinicaltrials.gov under registration number NCT06085885.

### Participants

The sample will comprise adults assessed as eligible to receive a unilateral cochlear implant through the National Health Service (NHS) in the UK. Participants will be recruited from the caseload of patients in participating cochlear implant service providers. Potential participants will be identified and approached by members of their clinical care team, either via searches of clinical databases or opportunistically during routine clinical appointments. Posters will be displayed in relevant clinical areas inviting patients to inquire about the study with their clinical care team or the study team. Potential participants will be provided with a participant information sheet and be invited to direct any questions at members of their clinical care team or the study team. Informed consent from patients who decide to take part will be taken online before data collection commences.

Secondary, non-clinical recruitment routes will include study advertisements via the NIHR Nottingham BRC newsletter, social media (e.g., NIHR Nottingham BRC Twitter, Facebook accounts) and other online advertisements, including professional and charity organisations (e.g., British Cochlear Implant Group, Tinnitus UK, National Cochlear Implant Users Association). However, potential participants will only be able to take part if they are receiving their treatment at one of the participating cochlear implantation service providers.

The inclusion criteria are as follows:

- Aged 18 years or older.
- Determined by clinical care team to be eligible for unilateral cochlear implantation and proceed to receive one.
- Sufficient written or spoken English to participate in study activities.
- Able to give informed consent.
- Did not previously receive a cochlear implant in either ear.
- Have access to computer or other suitable device with internet access to complete online study questionnaires.
- Able to give informed consent.

The exclusion criteria are as follows:

- Existing or previous cochlear implant user.
- No access to computer or other suitable device with internet access to complete online study questionnaires.
- Unable to complete study activities independently.

The study will recruit at least 50 participants with tinnitus pre-implantation. Participants will provide written informed consent ahead of participation. The study is powered on the Tinnitus Functional Index (TFI) as primary tinnitus outcome measure to determine meaningful changes in tinnitus outcomes following cochlear implantation. The minimum difference on the TFI has not been estimated in the present population and it is not possible to estimate the sample size directly. Previous data on the performance of the TFI in the general UK population suggest an average TFI score of 50.8 (standard deviation = 25.1), and a minimum difference of 17.9 points to improve tinnitus from being perceived as a moderate to a small problem–which could be considered as a meaningful change from a tinnitus severity category considered as bothersome to its lowest self-reported category [26]. Therefore, assuming the average and standard deviation estimates reported above, and a weak correlation of r = 0.3 between the pre- and post-implantation TFI scores, a conservative estimate based on a one-tailed t-test at a significance level of 0.05 suggests that a sample of 31 patients would be required to detect the minimum improvement on TFI that would be meaningful to patients. Considering a very conservative attrition rate of 38% [26], about 50 patients with tinnitus would be needed to detect a minimum reduction in TFI score post-implantation. This large, conservative, attrition rate has been used due to the new patient-led method of data collection using online questionnaires. Discontinued/withdrawn participants will be replaced until the minimum target of 50 participants is reached and recruitment of new participants enables following up for at least 3 months within the duration of the study.

### Study regimen

Data will be collecting using a range of standardised questionnaires, using the online JISC survey software (https://www.onlinesurveys.ac.uk). The following questionnaires will be administered:

#### Primary outcome measure

Tinnitus symptom severity5

- Tinnitus Functional Index (TFI) to assess the impact of tinnitus on the patients reporting tinnitus [27]. NICE has recommended the TFI for the assessment of tinnitus impact in adults [28], and changes on TFI will be used to address the primary objective of the study. The maximum overall score is 100, with higher scores indicating greater tinnitus symptom severity.

#### Secondary outcome measures

Tinnitus and hearing status

- Tinnitus case profiling questionnaire (ESiT-SQ) to comprehensively characterise tinnitus-related socio-demographic, health and lifestyle factors, sound tolerance, tinnitus presence and its characteristics and access to treatments in those who report having tinnitus [29]. This is a case history questionnaire without a numerical scoring system.
- Speech, Spatial and Qualities 12 (SSQ-12) to assess hearing function in everyday situations [30]. The SSQ has been found to be sensitive for detecting listening difficulties associated with changes in the severity of hearing loss in the general population [31, 32], and an efficient and sensitive measure of changes in self-reported hearing function in cochlear implant recipients [33]. Scores range from 0 to 120, with a higher score indicating better hearing ability.

Mental health

- Patient Health Questionnaire (PHQ-9) to assess the presence and severity of depressive symptoms [34]. The PHQ-9 is a standard diagnostic questionnaire widely used in both clinical settings and research studies, including assessments of depressive symptoms in patients with tinnitus [35, 36]. The PHQ-9 is scored from 0 to 27, with a higher score indicating more severe symptoms.
- Generalized Anxiety Disorder (GAD-7) to assess the presence and severity of anxiety symptoms [37]. The GAD-7 is a standard diagnostic questionnaire widely used in both clinical settings and research including studies assessing anxiety in the general tinnitus population [35], and cochlear implant recipients [24, 38]. The GAD is scored from 0 to 21, with higher scores indicating more severe anxiety.
- Insomnia Severity Index (ISI) to assess the presence, severity and nature of insomnia symptoms [39]. The ISI has been found to be a reliable measure for detecting clinically abnormal insomnia symptoms [40, 41]. It has been widely used in studies investigating insomnia in the general tinnitus population [35, 42, 43], cochlear implant recipients [17], and clinical trials evaluating effectiveness of interventions for insomnia [44]. The ISI is scored from 0 to 28, with higher scores indicating more severe insomnia.

Quality of life

- Two Quality of Life (QoL) questionnaires to assess changes in (a) hearing-related quality of life using the Health Utilities Index Mark 3 [45] and (b) health-related quality of life using the EuroQol EQ-5D-5L questionnaire [46]. Both questionnaires will be used as Health Utilities Index Mark 3 is used by NICE in recommendations for the provision of cochlear implantation in the UK [18], while EQ-5D is the preferred instrument by NICE to measure health-related quality of life with the ‘5L’ version supported for use in prospective clinical studies [47, 48]. The EQ-5D has been also shown to be sensitive to changes in tinnitus and recommended for evaluating interventions intended to alleviate tinnitus [23]. The EQ-5D-5L is scored from 0 to 120, with a lower score indicating worse health. The HUI-3 score ranges from 0.00 (death) to 1.00 (perfect health).

**Fig 1:**
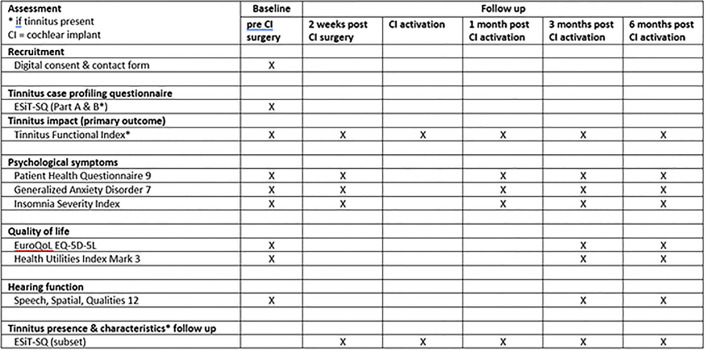
Schedule of online questionnaires.

Data will be collected at baseline, 2 weeks after cochlear implantation, immediately after the activation of the implant, and at 1, 3 and 6 months after activation. For an overview of which questionnaires will be administered at each timepoint, please refer to Fig 1.

### Planned analyses

Data analyses will be conducted by the study team with support from a medical statistician, using Microsoft Excel and SPSS Statistics. Statistical methods will be used to characterise the sample and evaluate changes in the severity of tinnitus and patient-specific factors before and after implantation. Data analyses will include descriptive statistics, omnibus tests (e.g., ANOVA) or non-parametric statistical methods where appropriate (e.g., Mann Whitney U test). If data permits, participants will be grouped according to their socio-demographic and relevant patient-specific characteristics, including the presence, severity and nature of tinnitus, hearing-related outcomes, psychological symptoms and factors related to cochlear implantation or device use. We will construct regression models to explore associations between the severity of tinnitus and existence/severity of psychological symptoms, socio-demographic variables, hearing-related outcomes, and quality of life in patients with and without tinnitus. Data from participants who do not complete the final follow-up will still be analysed. Datasets missing more than that will not be analysed.

## Discussion

Cochlear implantation is an effective intervention that restores useful aspects of hearing in those with severe to profound hearing loss or who are Deaf. It may also be effective in reducing tinnitus symptom severity. The purpose of this study is to characterise changes in tinnitus outcomes in adults with bilateral severe-to-profound hearing loss undergoing cochlear implantation to improve our understanding of cochlear implantation as a potential intervention for tinnitus in adults. Existing data is insufficient to predict the tinnitus-related effects of cochlear implantation, making it difficult for clinicians to advise and inform potential cochlear implant recipients in an evidence-based way. The findings will inform clinical decision-making as well as future large-scale studies towards identifying predictive factors of changes in tinnitus following cochlear implantation and proposing tinnitus-related candidacy criteria for cochlear implantation to alleviate tinnitus.

## Supporting information

S1 Checklist SPIRIT 2013 checklist: Recommended items to address in a clinical trial protocol and related documents*. (DOC)

S1 File (DOCX)
