# Supplementary material for: Evaluation of factors predicting tinnitus outcomes following cochlear implantation: Protocol for a prospective quasi-experimental study
Source: PLoS One. 2024 Jun 17;19(6):e0302790. doi: 10.1371/journal.pone.0302790 (PMC11182524; doi:10.1371/journal.pone.0302790)
Supplement: S1 File — (DOCX) [file pone.0302790.s002.docx]

**EVALUATION OF FACTORS PREDICTING TINNITUS OUTCOMES FOLLOWING COCHLEAR IMPLANTATION: PROTOCOL**

**Final v1.4**

**13DEC2023**

**Short title:** Tinnitus and Cochlear Implants

**Acronym:** TICIT

**IRAS Project ID:** 292855

**Study Sponsor:** University of Nottingham

**Sponsor reference:** 23004

**Funding Source:** NIHR Nottingham Biomedical Research Centre

Hearing Theme

Support for CI salary, essential equipment and consumables costs

Cochlear Europe Ltd.

Support for participant and clinical staff involvement, questionnaire licence fees, part-funding for CI and CO-I salary. Support in kind through technical expertise and study publicity.

Contact: Dr Anne Small

Email: asmall@cochlear.com

NIHR Nottingham BRC Hearing Theme PPI Grant

Support for patient representative’s involvement, lay reviews and piloting online questionnaires.

Contact: Dr Adele Horobin

Phone: 0115 823 2614

Email: [Adele.Horobin@nottingham.ac.uk](mailto:Adele.Horobin@nottingham.ac.uk)

British Tinnitus Association

Support in kind through involvement as public collaborator on the Study Team, and study publicity.

STUDY PERSONNEL AND CONTACT DETAILS

**Sponsor:** University of Nottingham

Contact name Mr Ali Alshukry

Research and Innovation

University of Nottingham
E-Floor, Yang Fujia Building

Jubilee Campus, Wollaton Road,

Nottingham NG8 1BB

sponsor@nottingham.ac.uk

**Chief investigator:** Dr Derek Hoare

Associate Professor in Hearing Sciences

Hearing theme lead, NIHR Nottingham Biomedical Research Centre

Ropewalk House

113 The Ropewalk

Nottingham

NG1 5DU

Phone: 0115 823 2600

Phone (direct): 0115 823 2630

Email: derek.hoare@nottingham.ac.uk

**Co-investigators:** Laura Merwood

Senior Audiologist

University of Southampton Auditory Implant Service

Highfield

Southampton

SO17 1BJ

Dr Magdalena Sereda

Senior Research Fellow

NIHR Nottingham Biomedical Research Centre

Ropewalk House

113 The Ropewalk

Nottingham

NG1 5DU

Phone: 0115 823 2626

Email: [Padraig.Kitterick@nottingham.ac.uk](mailto:Padraig.Kitterick@nottingham.ac.uk)

Professor Helen Cullington

Professorial Fellow

Clinical Scientist (Audiology)

University of Southampton

Highfield

Southampton

SO17 1BJ

Phone: 023 8059 7606

Email: [hec@isvr.soton.ac.uk](mailto:hec@isvr.soton.ac.uk)

**Collaborators:** Susan Johnson

Audiology Lead

Nottingham Auditory Implant Programme

Nottingham University Hospitals NHS Trust

Ropewalk House

113 The Ropewalk

Nottingham

NG1 5DU

Email: [Susan.Johnson@nuh.nhs.uk](mailto:Susan.Johnson@nuh.nhs.uk)

Josephine Dunster

Patient Representative

Email: jmdunster1@virginmedia.com

TBC

Chief Executive of the British Tinnitus Association

British Tinnitus Association

Phone: 0114 250 9933

Email: David@tinnitus.org.uk

Dr Adele Horobin

Patient and Public Involvement lead

School of Medicine

University of Nottingham

NG7 2RD

Email: Adele.Horobin@nottingham.ac.uk

**Study Coordinating Centre:** NIHR Nottingham Biomedical Research Centre Hearing Sciences

University of Nottingham

Ropewalk House

113 The Ropewalk

Nottingham

NG1 5DU

Phone: 0115 823 2600

# SYNOPSIS

| Title | Evaluation of factors predicting tinnitus outcomes following cochlear implantation |
| --- | --- |
| Acronym | TICIT |
| Short title | Tinnitus and Cochlear Implants |
| Chief Investigator | Dr Derek Hoare |
| Objectives | **Primary objective**  Determine whether there are meaningful changes in tinnitus outcomes following cochlear implantation in adults with bilateral severe-to-profound hearing loss.  **Secondary objectives**   1. Determine the prevalence, nature, and severity of tinnitus before cochlear implantation. 2. Determine the incidence, nature, and changes in severity of tinnitus following cochlear implantation. 3. Explore associations between tinnitus and changes in hearing, psychological health, cochlear implantation-related factors, and quality of life in cochlear implant recipients with and without tinnitus. |
| Study Configuration | Prospective, observational study comprising two elements:   1. Standard questionnaires to characterise changes in tinnitus, hearing, psychological health, and quality of life. |
| Setting | **Patient identification:**  Providers of cochlear implantation services on the NHS in the UK  **Standard questionnaires:**  Completed remotely by participants using a computer or other preferred electronic device.  **Clinical data extraction:**  Extracted from patient records by NHS staff into electronic study forms. |
| Sample size estimate | The study is powered on the Tinnitus Functional Index (TFI) as primary tinnitus outcome measure to determine meaningful changes in tinnitus outcomes following cochlear implantation. The minimum difference on the TFI has not been estimated in the present population and it is not possible to estimate the sample size directly. Previous data on the performance of the TFI in the general UK population suggest a very conservative sample size of 50 patients with tinnitus to detect a meaningful change in tinnitus on TFI and to account for potential attrition due to new, patient-led data collection method.  The secondary objectives include exploring new incidence of tinnitus post implantation and factors that may distinguish patients with and without tinnitus in this population. Therefore, patients without tinnitus undergoing cochlear implantation will be also recruited. Considering the uncertainty about the true prevalence of tinnitus in candidates for cochlear implantation and potential disruptions to the service due to Covid-19, recruitment will continue after reaching the minimum target of 50 patients with tinnitus to maximise the sensitivity of the study. |
| Number of participants | The study will recruit at least 50 participants with tinnitus. Recruitment will continue to recruit as many patients as possible within the duration of the study that will enable following up patients for at least 3 months. |
| Eligibility criteria | The sample will comprise adults determined as eligible to receive a unilateral cochlear implant on the National Health Service (NHS) in the United Kingdom (UK). Inclusion criteria  - 18 years or older. - Determined to be eligible for unilateral cochlear implantation. - Did not previously receive a cochlear implant in either ear. - Sufficient written or spoken English to participate in study activities. - Have access to internet or suitable device to complete online study questionnaires. - Able to give informed consent.  Exclusion criteria  - Significant difficulties preventing independent completion of study activities. |
| Description of interventions | This study involves no clinical interventions beyond cochlear implantation that will be already available to participants as part of their routine care pathway. Participants will undergo routine pre- and post-operative assessments as part of usual care.  Online questionnaires will include standard instruments used to assess:   - Tinnitus and its characteristics, related socio-demographic, lifestyle and health-related factors, and access to tinnitus care (ESiT-SQ) - Tinnitus impact (TFI) - Tinnitus-related psychological symptoms including anxiety (PHQ-9) depression (GAD-7) and insomnia (ISI). - Quality of life sensitive to changes in hearing (HUI-3), and health and tinnitus (EQ-5D-5L) - Hearing function (SSQ-12)   The schedule of questionnaires will follow the schedule of routine clinical appointments as part of the cochlear implantation care pathway including a pre-operative baseline assessment, and 5 follow up assessments after the surgery to receive the cochlear implant, after first cochlear implant activation appointment, and after the 1-, 3- and 6-month appointments post-activation. The baseline questionnaire will require about 40 min to complete and follow up questionnaires about 15-30 min. |
| Duration of study | December 2023 – April 2025 |
| Methods of analysis | Detailed analyses will be specified prospectively in a study analysis plan developed by the study team. Data analyses will be conducted by the study team with support from medical statisticians at the NIHR Nottingham BRC and/or the University of Nottingham. The software utilised for analyses may include Excel, SPSS, R or MATLAB. Mixed statistical methods will be used to characterise the sample and evaluate changes in the severity of tinnitus and patient-specific factors before and after implantation. Data analyses will include descriptive statistics, General/Generalised Linear Models (e.g., ANOVA, regression models) or non-parametric statistical methods where appropriate (e.g., Mann Whitney U test). |

# ABBREVIATIONS

BCIG British Cochlear Implant Group

BRC Biomedical Research Centre

BTA British Tinnitus Association

CI Chief Investigator overall

CRF Case Report Form

CRN Clinical Research Network

ESiT-SQ European School for Interdisciplinary Tinnitus Research – Screening
Questionnaire

EQ-5D-5L EuroQol, 5-Dimension, 5-Level

GAD Generalized Anxiety Disorder

GCP Good Clinical Practice

HUI-3 Health Utility Index Mark 3

ISI Insomnia Severity Index

NCIUA National Cochlear Implant Users Association

NHS National Health Service

NICE National Institute for Health and Care Excellence

NIHR National Institute for Health Research

PHQ Patient Health Questionnaire

PI Principal Investigator at a local centre

PIS Participant Information Sheet

REC Research Ethics Committee

R&D Research and Development department

SSQ Speech Spatial Qualities of Hearing Scale

TFI Tinnitus Functional Index

UoN University of Nottingham

# TABLE OF CONTENTS

[STUDY PERSONNEL AND CONTACT DETAILS 3](#_Toc77546136)

[SYNOPSIS 5](#_Toc77546137)

[ABBREVIATIONS 7](#_Toc77546138)

[TABLE OF CONTENTS 8](#_Toc77546139)

[STUDY BACKGROUND INFORMATION AND RATIONALE 10](#_Toc77546140)

[What is currently known? 10](#_Toc77546141)

[What is the problem? 10](#_Toc77546142)

[Is this study relevant to current policy and patient need? 11](#_Toc77546143)

[Why there is a need for this type of study? 11](#_Toc77546144)

[What is the rationale for the types of information to be collected? 12](#_Toc77546145)

[STUDY OBJECTIVES AND PURPOSE 13](#_Toc77546146)

[PURPOSE 13](#_Toc77546147)

[PRIMARY OBJECTIVE 14](#_Toc77546148)

[SECONDARY OBJECTIVES 14](#_Toc77546149)

[STUDY DESIGN 14](#_Toc77546150)

[STUDY CONFIGURATION 14](#_Toc77546151)

[STUDY MANAGEMENT 14](#_Toc77546152)

[DURATION OF THE STUDY AND PARTICIPANT INVOLVEMENT 15](#_Toc77546153)

[End of the Study 15](#_Toc77546154)

[SELECTION AND WITHDRAWAL OF PARTICIPANTS 15](#_Toc77546155)

[Recruitment 15](#_Toc77546156)

[Eligibility criteria 16](#_Toc77546157)

[Inclusion criteria 16](#_Toc77546158)

[Exclusion criteria 16](#_Toc77546159)

[Expected duration of participant participation 16](#_Toc77546160)

[Participant Withdrawal 16](#_Toc77546161)

[Informed consent 17](#_Toc77546162)

[STUDY REGIMEN 17](#_Toc77546163)

[Compliance 22](#_Toc77546164)

[Criteria for terminating the study 22](#_Toc77546165)

[ANALYSES 22](#_Toc77546166)

[Methods 22](#_Toc77546167)

[Sample size and justification 22](#_Toc77546168)

[ADVERSE EVENTS 23](#_Toc77546169)

[ETHICAL AND REGULATORY ASPECTS 23](#_Toc77546170)

[ETHICS COMMITTEE AND REGULATORY APPROVALS 24](#_Toc77546171)

[INFORMED CONSENT AND PARTICIPANT INFORMATION 24](#_Toc77546172)

[RECORDS 24](#_Toc77546173)

[Case Report Forms 24](#_Toc77546174)

[Source documents 25](#_Toc77546175)

[Direct access to source data / documents 25](#_Toc77546176)

[DATA PROTECTION 25](#_Toc77546177)

[QUALITY ASSURANCE & AUDIT 26](#_Toc77546178)

[INSURANCE AND INDEMNITY 26](#_Toc77546179)

[STUDY CONDUCT 26](#_Toc77546180)

[STUDY DATA 26](#_Toc77546181)

[RECORD RETENTION AND ARCHIVING 26](#_Toc77546182)

[DISCONTINUATION OF THE STUDY BY THE SPONSOR 27](#_Toc77546183)

[STATEMENT OF CONFIDENTIALITY 27](#_Toc77546184)

[PUBLICATION AND DISSEMINATION POLICY 27](#_Toc77546185)

[USER AND PUBLIC INVOLVEMENT 28](#_Toc77546186)

[STUDY FINANCES 29](#_Toc77546187)

[Funding source 29](#_Toc77546188)

[Participant stipends and payments 29](#_Toc77546189)

[SIGNATURE PAGES 30](#_Toc77546190)

[REFERENCES 31](#_Toc77546191)

# STUDY BACKGROUND INFORMATION AND RATIONALE

## What is currently known?

Unilateral cochlear implantation is a clinically- and cost-effective intervention to restore useful aspects of hearing function in adults with severe-to-profound hearing loss in both ears (Bond et al. 2009). Adult cochlear implant recipients have generally an awareness of environmental sounds (Ching et al. 2004), and a good understanding of conversational speech in quiet listening conditions (UK Cochlear Implant Study Group 2004). Adult candidates eligible to receive a cochlear implant often experience tinnitus, a perception of sounds in the ears or head that do not come from an external source (Baguley et al. 2013). Population-based studies suggest a prevalence of tinnitus among cochlear implantation candidates to be at least 50% (Pierzycki et al. 2016), but it may be as high as 80% (Baguley & Atlas 2007). Tinnitus has been also associated with psychological symptoms such as anxiety, depression and insomnia, which can have a significant impact on the patient and their quality of life (Tyler & Baker 1983; Langguth 2011). Recent systematic reviews suggest that cochlear implantation can alleviate tinnitus and the impact it may impose (Ramakers et al. 2015; Zenner et al. 2017; Peter et al. 2019; Assouly et al. 2020).

## What is the problem?

Systematic reviews have identified large heterogeneity in the existing evidence about tinnitus-related outcomes after cochlear implantation (Ramakers et al. 2015; Assouly et al. 2020). This heterogeneity has been partly associated with the variability in the data collection methods and the types of outcome measures used to assess tinnitus and related characteristics. Current efforts in tinnitus research focus on systematic data collection in large patient populations and the use of standard outcome measures to ensure consistency and characterising meaningful changes in tinnitus outcomes to support patient-specific clinical management of tinnitus (Cederroth et al. 2019; S. S. Smith et al. 2021). However, a prospective, systematic, and comprehensive collection of recommended tinnitus outcomes is not routinely conducted in patients with bilateral severe-to-profound hearing loss undergoing cochlear implantation. Therefore, the availability of high-quality evidence from large-scale, multicentre research studies is limited in this patient group.

The lack of consistent and comprehensive data also prevents dissociation of changes in tinnitus following implantation from the beneficial effects of implantation on hearing and quality of life (Summerfield et al. 2019). Crucially, it does not allow making reliable predictions about tinnitus outcomes following cochlear implantation, and thus supporting clinical decision making and changes in practice for the management of tinnitus in patients with bilateral severe-to-profound hearing loss (Ramakers et al. 2015; Ramakers et al. 2018; Kloostra et al. 2019; Dixon et al. 2020). Recent population-based studies and systematic reviews suggest that about 50% of cochlear implant recipients may still experience tinnitus after implantation which may be bothersome or lead to clinical anxiety, depression or insomnia symptoms (Andersson et al. 2009; Pierzycki et al. 2016; Pierzycki & Kitterick 2021). However, how much the different tinnitus-related symptoms contribute to the perceived tinnitus-related impact in different patient groups, when these symptoms are most likely to occur, and whether they require management during cochlear implantation care pathway is not clear. Moreover, a small proportion of patients has been found to develop or experience a worsening of their existing tinnitus after implantation (Ramakers et al. 2015). While findings from small observational studies suggest potential association between surgical, or pre- or post-operative psychological symptoms during auditory rehabilitation and the incidence or worsening of tinnitus and related symptoms (Todt et al. 2015; Bruggemann et al. 2017), the factors, or the stages of cochlear implantation care pathway at which these factors might influence tinnitus, remain unknown. Taken together, there is a need for systematic and comprehensive data to address the current evidence gap and to improve our understanding about meaningful changes in tinnitus and related characteristics in patients undergoing cochlear implantation. This data would also lay a foundation towards identifying predictive factors of tinnitus-related outcomes and large-scale studies evaluating the effectiveness of cochlear implantation as an intervention to alleviate tinnitus.

## Study relevance to current policy and patient need

The National Institute for Health and Care Excellence (NICE) published a guideline for the assessment and management of tinnitus (NICE 2020). The consultation conducted as part of the guideline development has identified a lack of high-quality evidence about the effectiveness of sound therapies and amplification devices, including cochlear implants, for the management of tinnitus in people with severe-to-profound hearing loss. Therefore, NICE has made a recommendation for further research to evaluate the clinical- and cost-effectiveness of these interventions to alleviate tinnitus in this population.

The NICE consultation has also identified an inequality in the management of tinnitus in people with bilateral, severe-to-profound hearing loss. This inequality stems from their inability to access standard care for hearing loss and tinnitus such as hearing aids and sound generators due to severity of their hearing loss. Cochlear implantation remains the most clinically- and cost-effective intervention in this patient group because it restores access to hearing and conversational speech when hearing aids are no longer adequate (NICE 2009; NICE 2019a). However, despite the growing number of studies suggesting that implantation can be also beneficial for tinnitus (Ramakers et al. 2015), tinnitus is currently not included in the recommendations for cochlear implantation (NICE 2019a).

The British Cochlear Implant Group (BCIG), a group of health care professionals and other parties interested in cochlear implant provision in the UK, established a Candidacy Working Group to review cochlear implant candidacy in the UK (Vickers et al. 2016). The review involved existing implant users, those unable to access cochlear implants and clinicians who provide implantation services. A consensus statement published on behalf of the BCIG, has recognised that cochlear implantation “can also be appropriate where it is for the alleviation of tinnitus” (BCIG Candidacy Working Group, 2017). This statement is in line with the management of tinnitus in those with profound hearing loss being identified as one of the top 10 treatment uncertainties and priorities for further research by patients with tinnitus and clinicians involved in their care within the James Lind Alliance Priority Setting Partnership (Hall et al. 2013). Therefore, all key stakeholders including NICE recognise the need for tinnitus interventions, provision of cochlear implantation, and further research in people with bilateral severe-to-profound hearing loss.

## The need for this type of study

NICE guidance and systematic reviews identify the lack of randomised controlled trials and high-quality prospective studies on the effectiveness of cochlear implantation for tinnitus in people with bilateral, severe-to-profound hearing loss. This evidence gap arises primarily from specific challenges about the feasibility of conducting clinical trials of cochlear implantation for the alleviation of tinnitus. As tinnitus is currently not included in the recommendations for cochlear implantation (NICE 2019a), it is not possible to fully randomise patients because implantation cannot be withheld from patients who are eligible to receive a cochlear implant based on the current candidacy criteria. On the other hand, a waiting list control design could be unethical due to potential negative effect of delaying cochlear implantation on patient outcomes (Kim et al. 2018). It is also not easy to blind patients and clinicians to this intervention. Therefore, tinnitus is typically assessed as a secondary measure as part of clinical trials investigating cochlear implantation as an intervention to restore useful aspects of hearing in people with severe-to-profound hearing loss. However, these trials do not collect data about the different symptoms contributing to the impact from tinnitus such as anxiety, depression, or insomnia. Thus, it is often not possible to fully characterise changes in tinnitus or dissociate them from changes in hearing-related problems following implantation from the available evidence.

The purpose of the present study is to systematically characterise changes in tinnitus outcomes which are currently not collected during the care pathway, to determine what changes in tinnitus outcomes are meaningful in patients undergoing cochlear implantation. Systematic data collection at different time points during the implantation care pathway will also provide estimates of prevalence, incidence of new and worsening tinnitus and access to tinnitus care, and characterise the factors associated with changes in tinnitus-related outcomes. This will also allow exploration of the utility of different outcomes as predictors of tinnitus changes and tinnitus-related cochlear implantation candidacy criteria in future studies. Moreover, the data will be used to explore associations between tinnitus and factors related to hearing, cochlear implantation, psychological wellbeing, and quality of life in cochlear implant recipients with and without tinnitus (Summerfield et al. 2019). The added value of the present study is the patient-led choice and collection of outcomes which will enable characterisation of their importance to patients. This in turn will allow establishing their adequacy for use in future studies evaluating the effectiveness of cochlear implantation for the alleviation of tinnitus.

## Rationale for the types of information to be collected

There is a large variability of prevalence figures in cochlear implantation candidates reported across studies suggesting the prevalence as high as 80% on average (Baguley & Atlas 2007; Andersson et al. 2009; Pan et al. 2009; Amoodi et al. 2011). This large variability is likely in part due to the use of different definitions of tinnitus across studies which highlights the need for a large-scale study using consistent and recommended definition of tinnitus to evaluate the scale of the problem (McCormack et al. 2016). Moreover, the majority of currently available evidence about tinnitus-related characteristics before implantation comes from small or retrospective studies (Ramakers et al. 2015), or those conducted in ‘atypical’ populations such as patients with single-sided deafness (Peter et al. 2019; Assouly et al. 2020). As a result, there is limited consistent evidence about the demographic, hearing and tinnitus-related characteristics, and access to tinnitus care by patients with bilateral severe-to-profound hearing loss who are the typical population of patients receiving cochlear implants in the UK (NICE 2019a). Therefore, the present study will collect comprehensive data on hearing, tinnitus and related symptoms using tinnitus-related measures recommended by NICE and in the academic literature (Hall et al. 2018; National Institute for Health and Care Excellence 2020) to assess the scale of the problem and the effect of cochlear implantation on tinnitus in this population.

Recent systematic reviews suggest that cochlear implantation can alleviate tinnitus and the burden it imposes on the patients (Ramakers et al. 2015; Zenner et al. 2017; Peter et al. 2019; Assouly et al. 2020). However, a lack of change, worsening and incidence of new tinnitus following cochlear implantation have also been reported in up to about 30%, 25% and 10% of patients with bilateral, severe-to-profound hearing loss, respectively (Ramakers et al. 2015). The factors responsible for the lack of change, worsening or incidence of newly developed tinnitus and its characteristics remain unclear. Several small, observational studies suggest a potential effect of surgical and psychological factors associated with undergoing cochlear implantation itself, or tinnitus and hearing-related pre-operative handicap, and recommended further research and systematic monitoring of patients during the implantation care pathway to evaluate the contribution of different factors to changes in tinnitus (Olze et al. 2012; Todt et al. 2015; Bruggemann et al. 2017; Kloostra et al. 2019). Moreover, recent studies suggest a potential effect of tinnitus and sound tolerance on the way cochlear implants are programmed and used (Pierzycki et al. 2019; Pierzycki & Kitterick 2021). Therefore, the present study will collect comprehensive data on hearing, tinnitus and related symptoms, cochlear implantation surgery, device use and programming around the time of routine pre- and post-operative clinical appointments. The data will be used to determine the incidence, nature, and influence of different cochlear implantation-related factors on changes in tinnitus after cochlear implantation.

The symptoms contributing to the perceived burden from tinnitus and the extent to which they are alleviated by cochlear implantation can vary between patients (Tyler & Baker 1983; Andersson et al. 2009; Pierzycki et al. 2016; Knopke et al. 2017; Kloostra et al. 2018; Gomersall et al. 2019; Pierzycki & Kitterick 2021; S. S. Smith et al. 2021). Overall, about 50% of CI users can still perceive tinnitus after cochlear implantation (Pierzycki et al. 2016), which can be distressing in about 25% of patients (Andersson et al. 2009), and associated with clinically abnormal anxiety (42%), depression (14%) and insomnia symptoms (41%) (Pierzycki & Kitterick 2021). Cochlear implant recipients may still experience tinnitus and related psychological symptoms in some situations such as when their implant is switched off at night-time (Chadha et al. 2009; Pierzycki et al. 2016), consistent with the fact that alleviation of tinnitus occurs primarily when cochlear implant is switched on and stimulating the auditory nerve (Zeng et al. 2011). However, while the impact of tinnitus after implantation has been associated with increased anxiety and depression (Andersson et al. 2009; Olze et al. 2011; Kloostra et al. 2015), the findings from prospective studies also suggested relatively small changes in psychological symptoms despite significant reduction in tinnitus-related distress after implantation (Olze et al. 2011; Bruggemann et al. 2017). Moreover, similar proportions of cochlear implant users (75%) and candidates for cochlear implantation (82%) report difficulties with sleep (Pierzycki et al. 2016).These findings are consistent with the existence of clinically abnormal anxiety, depression and insomnia symptoms in patients undergoing implantation who do not experience tinnitus (Pierzycki & Kitterick 2021), and highlight the need to characterise psychological symptoms in both cochlear implant recipients with and without tinnitus. The effect on psychological well-being may also be associated with the patient’s hearing loss (Gopinath et al. 2010; Nondahl et al. 2011). These psychological and hearing-related factors could in turn influence the patient’s quality of life following implantation (Olze et al. 2012; Summerfield et al. 2019). Therefore, the present study will also explore factors related to hearing, psychological wellbeing, and quality of life in recipients with and without tinnitus.

# STUDY OBJECTIVES AND PURPOSE

## PURPOSE

The purpose of the study is to characterise changes in tinnitus outcomes in adults with bilateral severe-to-profound hearing loss undergoing cochlear implantation to improve our understanding of cochlear implantation as an intervention for tinnitus in adults. The findings will inform future large-scale studies towards identifying predictive factors of changes in tinnitus following cochlear implantation and proposing tinnitus-related candidacy criteria for cochlear implantation to alleviate tinnitus.

## PRIMARY OBJECTIVE

Determine whether there are meaningful changes in tinnitus outcomes following cochlear implantation in adults with bilateral severe-to-profound hearing loss.

## SECONDARY OBJECTIVES

1. Determine the prevalence, nature, and severity of tinnitus before cochlear implantation.
2. Determine the incidence, nature, and changes in severity of tinnitus following cochlear implantation.
3. Explore associations between tinnitus and changes in hearing, psychological health, cochlear implantation-related factors, and quality of life in cochlear implant recipients with and without tinnitus.

# STUDY DESIGN

## STUDY CONFIGURATION

This will be a multicentre, prospective, observational study. Participants will be identified and recruited through participating cochlear implantation service providers from their patient caseload and through advertisements of public and professional organisations (e.g., BCIG, BTA, NCIUA), websites, and social media. Participants will undergo routine clinical assessments conducted by their cochlear implantation service providers as part of cochlear implantation care pathway. Research activities will involve completing standard questionnaires by participants online using a computer or other preferred electronic device.

## STUDY MANAGEMENT

NIHR Nottingham BRC will be the study co-ordinating centre. The project will be managed by the study team of Co-Investigators (Co-Is), clinicians, and patient and public collaborators led by the CI as listed below:

- Dr Derek Hoare, Research lead for tinnitus, lead for hearing theme, University of Nottingham (CI)
- Dr Magdalena Sereda, Senior Research Fellow, University of Nottingham (Co-I)
- Professor Helen Cullington, Professorial Fellow, University of Southampton, Clinical Scientist (Audiology), The University of Southampton Auditory Implant Service, Chair of the British Cochlear Implant Group, (Co-I)
- Susan Johnson, Audiology Lead, Nottingham Auditory Implant Programme, Nottingham University Hospitals NHS Trust (Collaborator)
- TBA, Chief Executive of the British Tinnitus Association (Collaborator)
- Josephine Dunster, Patient Representative, Cochlear implant recipient with experience of tinnitus (Collaborator)
- Dr Adele Horobin, Patient Involvement and Engagement Manager, NIHR Nottingham BRC (Collaborator)

The Chief Investigator has overall responsibility for the study and shall oversee all study management.

The data custodian will be the Chief Investigator.

## DURATION OF THE STUDY AND PARTICIPANT INVOLVEMENT

Study Duration: The study will open for recruitment in December 2023 and the final data will be collected before April 30^th^ 2025.

Participant Duration: We envisage an extended follow-up period of about 12 months from the time of first assessment at baseline. Considering the uncertainty about the true prevalence of tinnitus in candidates for cochlear implantation and potential disruptions to the service due to Covid-19, recruitment will continue beyond the minimum sample of 50 patients with tinnitus to recruit as many patients as possible within the duration of the study that will enable following up patients for at least 3 months.

Participants will take part in a baseline and five follow up online sessions consisting of multiple questionnaires. The baseline session will take around 45 minutes but will depend on how long the participant takes to complete each questionnaire. The frequency and duration of follow up sessions will decrease with time and will take around 15-30 minutes to complete.

### End of the Study

The end of the study will be the last assessment of the last recruited participant within the study duration.

## SELECTION AND WITHDRAWAL OF PARTICIPANTS

### Recruitment

Primary recruitment route will be from the caseload of patients in participating cochlear implantation service providers. This recruitment setting is most appropriate for this study as these services determine the eligibility to receive a unilateral cochlear implant.

Hospital interpreters and translator services will not be required for this study as participants will be required to have sufficient written or spoken English to complete online study questionnaires and give informed consent.

Potential participants will be identified and approached by members of staff considered to be members of the patient clinical care team. NHS staff will identify patients who are eligible to take part via searches of clinical databases or opportunistically during routine clinical appointments. Potential participants will be approached and informed about the study remotely via video conferencing and transcription software used for routine remote clinical appointments (e.g., Microsoft Teams), telephone, email, an invitation letter in the post as appropriate, or face-to-face if they will be attending the clinic. Remote contact will facilitate recruitment of eligible patients who may not be attending the clinic either due to awaiting surgery to receive a cochlear implant and potential burden of long-distance travel to the clinic, or other restrictions.

Approached patients will be provided with details of the CI and the study website. They will be asked to visit the study website and read the Participant Information Sheet (PIS) located on the website. They will be asked to contact the CI if they have any questions.

Participants will receive information regarding the study on the study website. Participants visiting the website will be asked to read the Participant Information Sheet (PIS) on the website before moving onto the online digital consent process. The contact details of the CI will be provided on the website and in invitation letters should the participants wish to discuss the study and their participation further or have any questions.

Posters will be displayed in the relevant clinical areas and will direct interested candidates to speak to the member of the cochlear implant team at the site, contact the CI or visit the study website for information about the study.

Secondary, non-clinical recruitment routes will include study advertisements via:

- NIHR Nottingham BRC newsletter
- Advertising on social media (e.g., NIHR Nottingham BRC Twitter, Facebook accounts)
- Online advertisements, including professional and charity organisations (e.g., BCIG, British Tinnitus Association, NCIUA)

However, potential participants will only be able to take part if their treating organisation is a research site(s) in the study.

Potential participants responding to advertisements will be asked to visit the study website, contact their clinic, or the CI via email to confirm their interest and ask any questions.

It will be explained to the potential participants that entry into the study is entirely voluntary and that their treatment and care will not be affected by their decision. It will also be explained that they can withdraw at any time, but attempts will be made to avoid this occurrence. In the event of their withdrawal it will be explained that their data collected so far cannot be erased and we will seek consent to use the data in the final analyses where appropriate.

### Eligibility criteria

The sample will comprise adults determined as eligible to receive a unilateral cochlear implant on the National Health Service (NHS) in the United Kingdom (UK).

### Inclusion criteria

- 18 years or older.
- Determined by clinical care team to be eligible for unilateral cochlear implantation and proceed to receive one.
- Sufficient written or spoken English to participate in study activities.
- Able to give informed consent.
- Did not previously receive a cochlear implant in either ear.
- Have access to internet or suitable device to complete online study questionnaires.
- Able to give informed consent.

### Exclusion criteria

- Existing or previous cochlear implant user.
- No access to computer or suitable device with internet to complete study activities.
- Unable to complete study activities independently.

### Expected duration of participant participation

Participating in the study will take 3-12 months.

### Participant Withdrawal

Participants may be withdrawn from the study either at their own request or at the discretion of the site PI under the guidance of the CI. Participants will be made aware that this will not affect their future care. Participants will be made aware (via the information sheet and consent form) that should they withdraw the data collected to date cannot be erased and may still be used in the final analysis.

Information regarding discontinuation or withdrawal of a participant will be recorded on the day of discontinuation/withdrawal in the study database. Reasons for discontinuation/withdrawal will be recorded if given. Discontinued/withdrawn participants will be replaced until the minimum target of 50 participants is reached and recruitment of new participants enables following up for at least 3 months within the duration of the study.

### Informed consent

All potential participants will be provided with a link to a secure study website Jisc by their clinician or the CI, after they have discussed the study with a member of the research team. Consent will be obtained online before completing questionnaires and extraction of relevant clinical data for the study from patient records. Consent will be completed by the potential participants alone or with the assistance of a suitably trained NHS staff as appropriate (e.g., via video conference). Signed consent will be defined as the participant entering their name, selecting a consent to each item on the consent from, and then submitting the form sent to their known contact details. PI or suitably trained NHS staff to whom consent has been delegated will be notified about digital consent and contact form being completed on the study website to verify the patient details on the contact form against the participating service records and sign the consent form. A welcome email will be then sent to the participant containing an electronic copy of the signed digital consent form, Participant Information Sheet, and the link to the study questionnaire. A copy of the signed digital consent and contact form will be stored in the electronic study forms available to view for the site PI and suitably trained NHS staff at the participating site(s). The consent will be available to print to file in the participant’s medical notes if needed.

Should there be any subsequent amendment to the protocol which might affect participation in the study, continuing consent will be obtained using an amended digital consent form which will be signed by the participant.

## STUDY REGIMEN

The study comprises two stages: a pre-operative baseline and post-operative follow up. The study will follow the steps of the cochlear implantation care pathway as shown in Figure.1. Research assessments will involve standard questionnaires completed online by study participants.

**Online questionnaires**

Participants will be asked to complete the baseline (pre-operative) questionnaire after digital consent is obtained. Participants will be asked to complete the questionnaires as soon as they can and within 2 weeks from receiving the email with the link to the questionnaire. Participants will be asked to repeat the baseline questionnaire if they are still awaiting their cochlear implantation surgery after 3 months since the first baseline questionnaire completion. Repeat baseline assessments will be optional. Participants will receive a welcome email with a copy of the signed digital consent, information on how to access the questionnaire and study website, withdraw from the study should they no longer wish to take part, and contact details of the CI should they have any questions.

Following surgery to receive their cochlear implant, participants will be asked to complete follow up questionnaires at five time points: 2 weeks after the surgery, after their first cochlear implant activation appointment, and after appointments at about 1, 3 and 6 months post implant activation (Fig. 1). These time points have been chosen to align with routine appointments in the post-operative care pathway. As such, their timing may vary according to the scheduling of those routine appointments. Participants will be sent emails with links to follow up questionnaires and asked to complete the questionnaires as soon as they can and within 2 weeks from the date their surgery and clinical appointments have taken place as indicated in the electronic study log by trained NHS staff for data extraction and entry at the site(s).

The study questionnaires will comprise a series of standard questionnaires completed according to the schedule described in Table 1. The questionnaires will be delivered online and no identifiable information will be requested (standard format including identifiers included in the application for example only). The questionnaires will include:

- **Tinnitus case profiling questionnaire (ESiT-SQ)** to comprehensively characterise tinnitus-related socio-demographic, health and lifestyle factors, sound tolerance, tinnitus presence and its characteristics and access to treatments in those who report having tinnitus (Genitsaridi et al. 2019).The full questionnaire will be completed at baseline, while a modified subset of questions will be used at follow up to establish the presence of tinnitus and changes in related characteristics since previous assessment.
- **Tinnitus Functional Index (TFI)** to assess the impact of tinnitus on the patients reporting tinnitus (Meikle et al. 2012). NICE has recommended the TFI for the assessment of tinnitus impact in adults (NICE 2020), and changes on TFI will be used to address the primary objective of the study. Participants who do not experience tinnitus will not complete this questionnaire.

.

- **Patient Health Questionnaire (PHQ-9)** to assess the presence and severity of depressive symptoms (Kroenke et al. 2001). The PHQ-9 is a standard diagnostic questionnaire widely used in both clinical settings and research studies, including assessments of depressive symptoms in patients with tinnitus (Wallhausser-Franke et al. 2017; Aazh & Moore 2019).
- **Generalized Anxiety Disorder (GAD-7)** to assess the presence and severity of anxiety symptoms (Spitzer et al. 2006). The GAD-7 is a standard diagnostic questionnaire widely used in both clinical settings and research including studies assessing anxiety in the general tinnitus population (Aazh & Moore 2019), and cochlear implant recipients (Olze et al. 2011; Bruggemann et al. 2017).
- **Insomnia Severity Index (ISI)** to assess the presence, severity and nature of insomnia symptoms (Bastien et al. 2001). The ISI has been found to be a reliable measure for detecting clinically abnormal insomnia symptoms (Smith & Wegener 2001; 2003; Gagnon et al. 2013). It has been widely used in studies investigating insomnia in the general tinnitus population (Miguel et al. 2014; Cronlein et al. 2016; Aazh & Moore 2019), cochlear implant recipients (Pierzycki & Kitterick 2021), and clinical trials evaluating effectiveness of interventions for insomnia (Curtis et al. 2021).
- **Two Quality of Life (QoL) questionnaires** to assess changes in (a) hearing-related quality of life using the Health Utilities Index Mark 3 (Feeny et al. 1995) and (b) health-related quality of life using the EuroQol EQ-5D-5L questionnaire (Herdman et al. 2011). Both questionnaires will be used as Health Utilities Index Mark 3 is used by NICE in recommendations for the provision of cochlear implantation in the UK (NICE 2019a), while EQ-5D is the preferred instrument by NICE to measure health-related quality of life with the ‘5L’ version supported for use in prospective clinical studies (NICE 2013, 2019b). The EQ-5D has been also shown to be sensitive to changes in tinnitus and recommended for evaluating interventions intended to alleviate tinnitus (Summerfield et al. 2019).
- **Speech, Spatial and Qualities 12 (SSQ-12)** to assess hearing function in everyday situations (Noble et al. 2013). The SSQ has been found to be sensitive for detecting listening difficulties associated with changes in the severity of hearing loss in the general population (Gatehouse & Noble 2004; Singh & Pichora-Fuller 2010), and an efficient and sensitive measure of changes in self-reported hearing function in cochlear implant recipients (Wyss et al. 2019).

| Figure 1. Study flow chart. |
| --- |
| 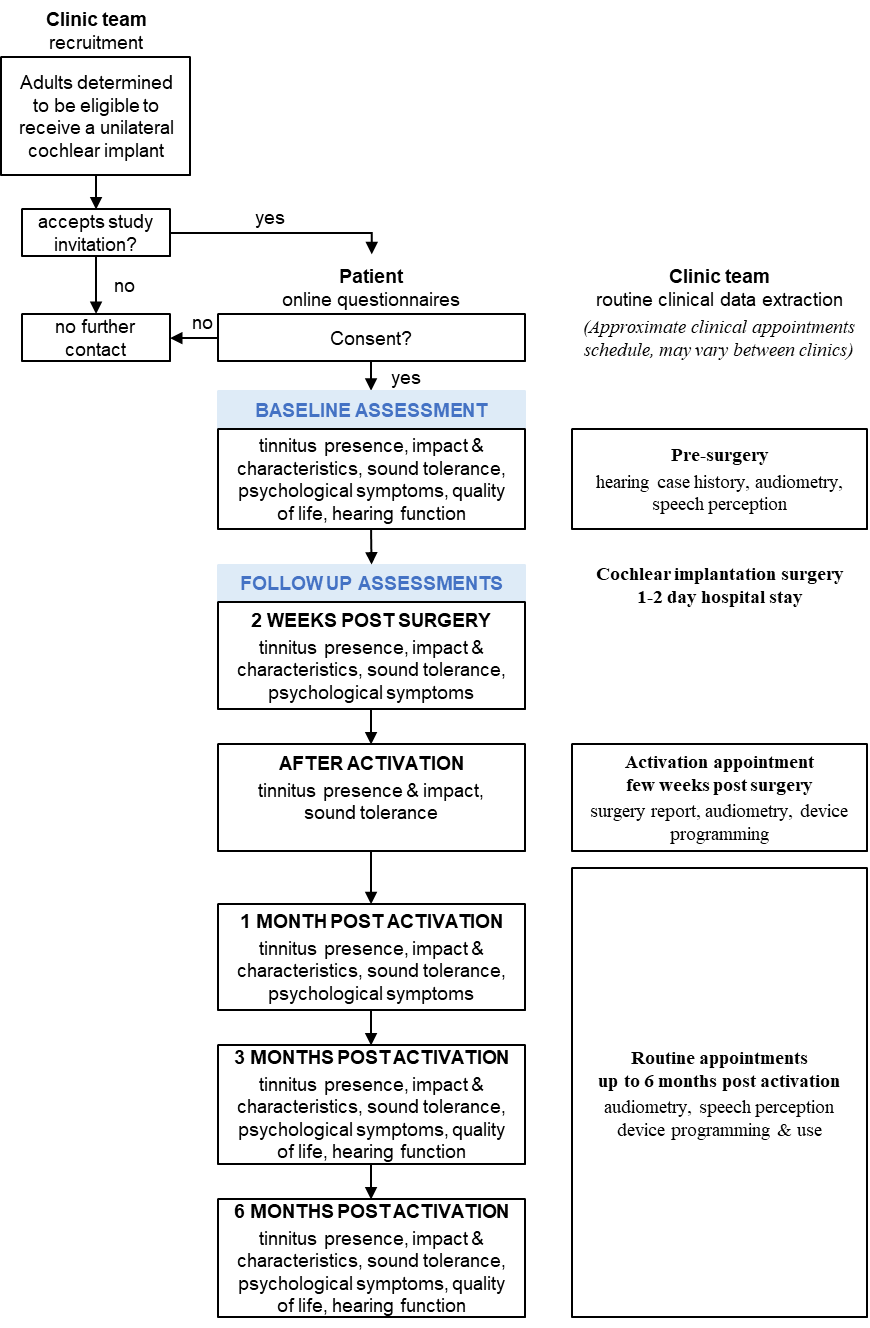 |

Table 1. Schedule of online questionnaires.

| **Assessment**  * if tinnitus present  CI = cochlear implant | **Baseline** | **Follow up** | | | | |
| --- | --- | --- | --- | --- | --- | --- |
|  | **pre CI surgery** | **2 weeks post CI surgery** | **CI activation** | **1 month post CI activation** | **3 months post CI activation** | **6 months post CI activation** |
| **Recruitment** |  |  |  |  |  |  |
| Digital consent & contact form | X |  |  |  |  |  |
|  |  |  |  |  |  |  |
| **Tinnitus case profiling questionnaire** |  |  |  |  |  |  |
| ESiT-SQ (Part A & B*) | X |  |  |  |  |  |
| **Tinnitus impact (primary outcome)** |  |  |  |  |  |  |
| Tinnitus Functional Index* | X | X | X | X | X | X |
|  |  |  |  |  |  |  |
| **Psychological symptoms** |  |  |  |  |  |  |
| Patient Health Questionnaire 9 | X | X |  | X | X | X |
| Generalized Anxiety Disorder 7 | X | X |  | X | X | X |
| Insomnia Severity Index | X | X |  | X | X | X |
|  |  |  |  |  |  |  |
| **Quality of life** |  |  |  |  |  |  |
| EuroQoL EQ-5D-5L | X |  |  |  | X | X |
| Health Utilities Index Mark 3 | X |  |  |  | X | X |
|  |  |  |  |  |  |  |
| **Hearing function** |  |  |  |  |  |  |
| Speech, Spatial, Qualities 12 | X |  |  |  | X | X |
|  |  |  |  |  |  |  |
| **Tinnitus presence & characteristics* follow up** |  |  |  |  |  |  |
| ESiT-SQ (subset) |  | X | X | X | X | X |

### Compliance

Due to observational nature of study, compliance will not be monitored. To minimise non-compliance participants will be encouraged to complete questionnaires as soon as possible and within 2 weeks of sending the questionnaire link before it expires. Two automated reminders will be sent within the 2-week period (after day 5 and 10) if questionnaires are not completed by that time.

### Criteria for terminating the study

No stopping points will be defined other than the study end date.

# ANALYSES

### Methods

Data analyses will be conducted by the study team with support from medical statisticians at the NIHR Nottingham BRC and/or the University of Nottingham. The software utilised for analyses may include Excel, SPSS, R or MATLAB. The data generated from statistical analysis will be stored and backed-up on the University of Nottingham servers.

Detailed analyses will be specified prospectively in the study analysis plan developed by the study team. Mixed statistical methods will be used to characterise the sample and evaluate changes in the severity of tinnitus and patient-specific factors before and after implantation. Data analyses will include descriptive statistics, General/Generalized Linear Models (e.g. ANOVA, regression models) or non-parametric statistical methods where appropriate (e.g. Mann Whitney U test). Participants may be grouped according to their socio-demographic and relevant patient-specific characteristics, including the presence, severity and nature of tinnitus, hearing-related outcomes, psychological symptoms and factors related to cochlear implantation or device use. We will seek to construct regression models to explore associations between the severity of tinnitus and existence/severity of psychological symptoms, socio-demographic variables, hearing-related outcomes and quality of life in patients with and without tinnitus (Pierzycki et al. 2016; Pierzycki & Kitterick 2021).

### Sample size and justification

The primary objective of the study is to determine whether there are meaningful changes in tinnitus outcomes following cochlear implantation in adults with bilateral severe-to-profound hearing loss. Therefore, the study is powered on the Tinnitus Functional Index (TFI) as primary tinnitus outcome measure. The minimum difference on the TFI has not been estimated in the present population and it is not possible to estimate the sample size directly. Previous data on the performance of the TFI in the general UK population suggest an average TFI score of 50.8 (standard deviation = 25.1), and a minimum difference of 17.9 points to improve tinnitus from being perceived as a moderate to a small problem – which could be considered as a meaningful change from a tinnitus severity category considered as bothersome to its lowest self-reported category (Fackrell et al. 2018). Therefore, assuming the average and standard deviation estimates reported above, and a weak correlation of r = 0.3 between the pre- and post-implantation TFI scores, a conservative estimate based on a one-tailed t-test at a significance level of 0.05 suggests that a sample of 31 patients would be required to detect the minimum improvement on TFI that would be meaningful to patients. Considering a very conservative attrition rate of 38% (Fackrell et al. 2018), about 50 patients with tinnitus would be needed to detect a minimum reduction in TFI score post-implantation. This large, conservative, attrition rate has been used due to the new patient-led method of data collection using online questionnaires.

The secondary objectives include exploring new incidence of tinnitus post implantation and factors such as psychological well-being that may distinguish patients with and without tinnitus in this population. Therefore, patients without tinnitus undergoing cochlear implantation will be also recruited. Estimates of prevalence of tinnitus in this population vary with population-based studies and reviews suggesting a prevalence of at least 50% and as high as 80% on average (Baguley & Atlas 2007; Pierzycki et al. 2016). Considering the uncertainty about the true prevalence of tinnitus in candidates for cochlear implantation and potential disruptions to the service due to Covid-19, recruitment will continue after reaching the minimum target of 50 patients with tinnitus to maximise the sensitivity of the study. Recruitment will continue to recruit as many patients as possible within the duration of the study that will enable following up patients for at least 3 months.

# ADVERSE EVENTS

The occurrence of an adverse event specifically as a result of participation within this study is not expected and no adverse event data will be collected.

# ETHICAL AND REGULATORY ASPECTS

Participants and NHS staff will follow COVID-19 safety procedures and infection control measures set out by the cochlear implantation service providers in line with the guidance from Public Health England.

There are no specific risks associated with the research activities. Study questionnaires will be completed by participants remotely, outside of clinical appointments. The frequency and the time required to complete the questionnaires will reduce with time and experience and will not place an appreciable burden on participants or affect their care. There will be no direct benefits to participants from taking part in the study. Voucher payments will be paid to participants as an inconvenience allowance for their time completing study assessments at their final assessment. However, the results will help to inform the design of future trials evaluating the effectiveness of cochlear implantation for the alleviation of tinnitus, which could provide benefits to other patients in the future.

The study will use standard questionnaires and monitoring questions used in research and clinical setting that ask participants about tinnitus and related case history, hearing and listening abilities and psychological well-being. The questionnaires will not provide participants with assessment scores or clinical diagnoses. However, the questionnaires may potentially raise the awareness of participant’s tinnitus, related health conditions, and psychological symptoms. Contact details to British Tinnitus Association help line will be provided in the participant information sheet should the participant wish to seek advice about their tinnitus and related symptoms. Seeking help for tinnitus and access to tinnitus interventions will be also monitored during the study as part of the questionnaire to characterise their potential influence on changes in tinnitus and related outcomes.

The system will use secure data capture methods (e.g. dedicated questionnaire invites) and electronic study forms with password-protected access. The data will be anonymised against the participant identification code and stored on a secure dedicated web server.

## ETHICS COMMITTEE AND REGULATORY APPROVALS

The study will not be initiated before the protocol, consent forms and participant information sheets have received approval / favourable opinion from the Research Ethics Committee (REC), the respective National Health Service (NHS) or other healthcare provider’s Research & Development (R&D) department, and the Health Research Authority (HRA) if required. Should a protocol amendment be made that requires REC approval, the changes in the protocol will not be instituted until the amendment and revised informed consent forms and participant information sheets (if appropriate) have been reviewed and received approval / favourable opinion from the REC and R&D departments. A protocol amendment intended to eliminate an apparent immediate hazard to participants may be implemented immediately providing that the REC are notified as soon as possible and an approval is requested. Minor protocol amendments only for logistical or administrative changes may be implemented immediately; and the REC will be informed.

The study will be conducted in accordance with the ethical principles that have their origin in the Declaration of Helsinki, 1996; the principles of Good Clinical Practice and the UK Department of Health Policy Framework for Health and Social Care, 2017.

## INFORMED CONSENT AND PARTICIPANT INFORMATION

The process for obtaining participant informed consent will be in accordance with the REC guidance, and Good Clinical Practice (GCP) and any other regulatory requirements that might be introduced. The participant shall both sign and date the digital Consent Form before the they can participate in the study.

The participant will receive an electronic copy of the signed and dated forms and the original will be retained in the password-protected electronic study records. The electronic copy of the consent will be available to access and print by suitably trained NHS staff at the site to be filed in the participant’s medical notes if needed, and a signed and dated note made in the notes that informed consent was obtained for the study.

The decision regarding participation in the study is entirely voluntary. The investigator or their nominee shall emphasise to them that consent regarding study participation may be withdrawn at any time without penalty or affecting the quality or quantity of their future medical care, or loss of benefits to which the participant is otherwise entitled. No study-specific interventions will be done before informed consent has been obtained.

The investigator will inform the participant of any relevant information that becomes available during the study, and will discuss with them, whether they wish to continue with the study. If applicable they will be asked to sign revised consent forms.

If the Consent Form is amended during the study, the investigator shall follow all applicable regulatory requirements pertaining to approval of the amended Consent Form by the REC and use of the amended form (including for ongoing participants).

## RECORDS

### Case Report Forms

Each participant will be assigned a study identity code number, for use on any printed documents and the electronic database. Printed documents and database will also use their initials (of first and last names separated by a hyphen or a middle name initial when available) and date of birth (yy/mm/dd) to generate a unique participant identifier for the digital contact form: e.g., Jane Smith 01/01/1980 will be assigned TICIT.J-S.800101. The unique identifier will be used for electronic participant identification lists for suitably trained NHS staff at study site(s) to identify participants, keep a log of appointments to send out study and questionnaire invitations and collect information about discontinued/withdrawn participants and their reasons if provided. Information about recruitment including the numbers of identified potential participants, invitations sent, and recruited/discontinued/withdrawn patients, will be available to CI to understand recruitment efforts, monitor success against milestones and identify potential support if needed.

Study forms will be treated as confidential documents and held securely in accordance with regulations. Participants will complete digital contact forms for the investigator to make a separate confidential record of the participant’s name, date of birth, NHS number, address, email, phone number and Participant Study Number, to permit identification of all participants enrolled in the study, and in case additional follow-up is required. Study forms shall be restricted to those personnel approved by the Chief or local Investigator and recorded as such in the study records.

If for whatever reason online forms are printed, they will be stored securely, and annotated using black ballpoint pen. Errors shall be lined out but not obliterated by using correction fluid and the correction inserted, initialled, and dated.

The Chief or local Investigator shall sign a declaration ensuring accuracy of data recorded in the CRF.

### Source documents

Source documents shall be filed at the study coordinating centre and may include but are not limited to, consent forms, study records, and questionnaires. . A CRF may also completely serve as its own source data. Only study staff shall have access to study documentation other than the regulatory requirements listed below.

### Direct access to source data / documents

The CRF and all source documents shall always be made available for review by the Chief Investigator, Sponsor’s designee, and inspection by relevant regulatory authorities.

## DATA PROTECTION

All study staff and investigators will endeavour to protect the rights of the study’s participants to privacy and informed consent, and will adhere to the Data Protection Act, 2018. The study forms will only collect the minimum required information for the purposes of the study. Any printed study materials will be held securely, in a locked room, or locked cupboard or cabinet. Access to the information will be limited to the study staff and investigators and any relevant regulatory authorities (see above). Computer held data including the study database will be held securely and password protected. All data will be stored on University of Nottingham secure systems. Access will be restricted by user identifiers and passwords (encrypted using a one-way encryption method).

Information about the study in the participant’s medical records / hospital notes will be treated confidentially in the same way as all other confidential medical information.

Electronic data will be backed up every 24 hours to both local and remote media in encrypted format.

# QUALITY ASSURANCE & AUDIT

## INSURANCE AND INDEMNITY

Insurance and indemnity for clinical study participants and study staff is covered within the NHS Indemnity Arrangements for clinical negligence claims in the NHS, issued under cover of HSG (96)48. There are no special compensation arrangements, but study participants may have recourse through the NHS complaints procedures.

The University of Nottingham as research Sponsor indemnifies its staff with both public liability insurance and clinical trials insurance in respect of claims made by research subjects.

## STUDY CONDUCT

Study conduct may be subject to systems audit for inclusion of essential documents; permissions to conduct the study; CVs of study staff and training received; local document control procedures; consent procedures and recruitment logs and adherence to procedures defined in the protocol (e.g., inclusion / exclusion criteria, timeliness of visits).

The Study Coordinator/Academic Supervisor, or where required, a nominated designee of the Sponsor, shall carry out a site-systems audit at least yearly, and an audit report shall be made.

## STUDY DATA

Monitoring of study data shall include confirmation of informed consent; source data verification; data storage and data transfer procedures; local quality control checks and procedures, back-up and disaster recovery of any local databases and validation of data manipulation. The Chief Investigator shall carry out monitoring of study data as an ongoing activity.

Entries on study forms will be verified by inspection against the source data. A sample of study forms (10% or as per the study risk assessment) will be checked on a regular basis for verification of all entries made. In addition the subsequent capture of the data on the study database will be checked. Where corrections are required these will carry a full audit trail and justification.

Study data and evidence of monitoring and systems audits will be made available for inspection by the REC as required.

## RECORD RETENTION AND ARCHIVING

In compliance with the ICH/GCP guidelines, regulations and in accordance with the University of Nottingham Code of Research Conduct and Research Ethics, the Chief or local Principal Investigator will maintain all records and documents regarding the conduct of the study. These will be retained for at least 7 years or for longer if required. If the responsible investigator is no longer able to maintain the study records, a second person will be nominated to take over this responsibility.

The study documents held by the Chief Investigator on behalf of the Sponsor shall be finally archived at secure archive facilities at the University of Nottingham. This archive shall include all anonymised audio recordings, study databases and associated meta-data encryption codes.

## DISCONTINUATION OF THE STUDY BY THE SPONSOR

The Sponsor reserves the right to discontinue this study at any time for failure to meet expected enrolment goals, for safety or any other administrative reasons. The Sponsor shall take advice as appropriate in making this decision.

## STATEMENT OF CONFIDENTIALITY

Individual participant medical or personal information obtained in this study are considered confidential and disclosure to third parties is prohibited with the exceptions noted above.

Participant confidentiality will be further ensured by utilising identification code numbers to correspond to treatment data in the computer files.

Such medical information may be given to the participant’s medical team and all appropriate medical personnel responsible for the participant’s welfare.

If information is disclosed during the study that could pose a risk of harm to the participant or others, the researcher will discuss this with the CI and where appropriate report accordingly.

Data generated in this study will be available for inspection on request by the participating physicians, the University of Nottingham representatives, the REC, local R&D Departments, and the regulatory authorities.

# PUBLICATION AND DISSEMINATION POLICY

Participants will not be identified in any publications. After completion of the study a final study report will be prepared for submission to the REC within one year of closing. Final study report may be obtained from the CI upon request.

The study team will develop the publication and dissemination strategy for publications resulting from this research, including peer-reviewed publication, seminars or conferences, and the world-wide web. Data from the study may be also shared anonymously with other researchers outside of the research team to support other research in the future, and consent for sharing will be sought from the participant.

Funding and supporting bodies will be acknowledged in publications. Interim reports and dissemination materials will be provided to the study funders (i.e. Cochlear Ltd. and the Nottingham BRC Hearing Theme PPI Steering Group) as appropriate. The funders will not have an influence on the strategy and content of the dissemination materials. Regular study updates will be posted on social media by the CI or their nominee.

Publications resulting from the research will be made fully available through Gold Open Access publication. A lay summary report will be prepared in collaboration with the patient, public and PPI manager members of the Study Team (Dunster, Horobin, BTA) and sent to participants and stakeholder organisations (e.g., BCIG, BTA, NCIUA).

# USER AND PUBLIC INVOLVEMENT

This study design and protocol have been developed by the CI with support of Co-Is and collaborator members of the Study Team. The contributions included providing expertise on the clinical population, study objectives, choice of outcomes and study design (all protocol contributors). Clinician and patient collaborators have provided expertise on cochlear implant service delivery and patient experiences including those related to Covid-19 (Johnson, Cullington, Dunster). The protocol and study design have also been informed by the findings and direct reports from cochlear implant recipients with and without tinnitus in previous small-scale exploratory studies at the Nottingham Auditory Implant Programme. This information supported the choice of specific tinnitus-related outcomes, questionnaires, and study regimen.

Patients and public collaborators have been actively involved in the protocol development by providing expertise about support for people with tinnitus (Stockdale), and participation and contribution to research studies from the public (Dunster, Horobin). The input from the public (BTA) and the patient with experience of tinnitus (Dunster) at the design stage has helped to establish the relevance and importance of different outcomes to patients and potential burden from participation in the study. The contributions also included ways of integrating patient involvement into research so that it is appropriate, feasible, and likely to have impact on design of future large-scale studies to evaluate the effectiveness of cochlear implantation for tinnitus and inform changes to clinical practice. Their input has informed the types of outcome measures to be used in the study, patient information materials, the number and intervals at which patients will be asked to complete study assessments.

investigators met regularly with the members of the NIHR Nottingham BRC Hearing Theme PPI Steering Group during the study development period to seek input about various aspects of the study rationale and design. The Group has provided feedback and suggestions on the study design and patient engagement and involvement, including the ways of approaching potential participants, methods of remote data collection to reduce the burden on the patient and sustain their engagement, as well as payments and incentives to participants for their participation in the study. These suggestions have been implemented into the study design and protocol. The Steering Group has also made suggestions about the formation and membership of the Study Team by inclusion of the public and patient representatives (Dunster, BTA), and the then BRC PPI/E Manager to facilitate and support their involvement and that of study participants (Horobin). As part of a PPI funding application, the Steering Group has also reviewed the proposals for the nature of involvement and reimbursement to the patient representative for their involvement in Study Team activities. The Group has also suggested the inclusion and provided support for independent lay reviewers to review the study materials to ensure they are accessible to lay audience and piloting the online study questionnaires. The Group advised on the strategy for disseminating and assessing the impact of PPI on the study. The CI obtained guidance about PPI-related activities and patient involvement during design and will continue meeting with the Group throughout the duration of the study.

The study design and draft of the protocol were discussed and reviewed by members of the Nottingham Hearing Research Advisory Group (RAG) made up of clinician experts in audiology and delivery of hearing related research, including CRN representatives. This was to improve methodology for patient identification and recruitment, data collection, ensure clarity of the protocol, and advise on ways of supporting delivery of the study to successful completion. The CI will consult the members of the Group throughout the duration of the study period should any issues arise.

# STUDY FINANCES

### Funding source

This study is funded by NIHR Nottingham BRC through part salary to CI (Hoare) and Co-I (Sereda) and running expenses. Cochlear Ltd. provided support for clinicians and participants involved in the study, questionnaire licenses, and funding for 1x open access article processing charge. NIHR Nottingham BRC Hearing Theme PPI Steering Group supported the involvement of the patient representative, lay reviews of the study documentation and piloting of online questionnaires.

[Section removed for purpose of publication]

#

# SIGNATURE PAGES

[Section removed for purpose of publication]

# REFERENCES

Aazh, H. & Moore, B. C. J. (2019). Tinnitus loudness and the severity of insomnia: A mediation analysis. *Int J Audiol, 58*, 208-212.

Amoodi, H. A., Mick, P. T., Shipp, D. B., Friesen, L. M., Nedzelski, J. M., Chen, J. M., et al. (2011). The effects of unilateral cochlear implantation on the tinnitus handicap inventory and the influence on quality of life. *The Laryngoscope, 121*, 1536-1540.

Andersson, G., Freijd, A., Baguley, D. M., Idrizbegovic, E. (2009). Tinnitus distress, anxiety, depression, and hearing problems among cochlear implant patients with tinnitus. *J Am Acad Audiol, 20*, 315-319.

Assouly, K. K. S., van Heteren, J. A. A., Stokroos, R. J., Stegeman, I., Smit, A. L. (2020). Cochlear implantation for patients with tinnitus – A systematic review. In *Progress in Brain Research*: Elsevier.

Baguley, D. M. & Atlas, M. D. (2007). Cochlear implants and tinnitus. *Progress in Brain Research, 166*, 347-355.

Baguley, D. M., McFerran, D., Hall, D. A. (2013). Tinnitus. *Lancet, 382*, 1600-1607.

Bastien, C. H., Vallieres, A., Morin, C. M. (2001). Validation of the Insomnia Severity Index as an outcome measure for insomnia research. *Sleep Med, 2*, 297-307.

Bond, M., Mealing, S., Anderson, R., Elston, J., Weiner, G., Taylor, R. S., et al. (2009). The effectiveness and cost-effectiveness of cochlear implants for severe to profound deafness in children and adults: a systematic review and economic model. *Health Technology Assessment, 13*, 1-330.

British Cochlear Implant Group Candidacy Working Group. (2017). Consensus statement on candidacy for cochlear implantation. from <https://www.cicandidacy.co.uk>.

Bruggemann, P., Szczepek, A. J., Klee, K., Grabel, S., Mazurek, B., Olze, H. (2017). In patients undergoing cochlear implantation, psychological burden affects tinnitus and the overall outcome of auditory rehabilitation. *Frontiers in Human Neuroscience, 11*, 226.

Cederroth, C. R., Gallus, S., Hall, D. A., Kleinjung, T., Langguth, B., Maruotti, A., et al. (2019). Editorial: Towards an understanding of tinnitus heterogeneity. *Front Aging Neurosci, 11*, 53.

Chadha, N. K., Gordon, K. A., James, A. L., Papsin, B. C. (2009). Tinnitus is prevalent in children with cochlear implants. *International Journal of Pediatric Otorhinolaryngology, 73*, 671-675.

Ching, T. Y., Incerti, P., Hill, M. (2004). Binaural benefits for adults who use hearing aids and cochlear implants in opposite ears. *Ear and Hearing, 25*, 9-21.

Cronlein, T., Langguth, B., Pregler, M., Kreuzer, P. M., Wetter, T. C., Schecklmann, M. (2016). Insomnia in patients with chronic tinnitus: Cognitive and emotional distress as moderator variables. *Journal of Psychosomatic Research, 83*, 65-68.

Curtis, F., Laparidou, D., Bridle, C., Law, G. R., Durrant, S., Rodriguez, A., et al. (2021). Effects of cognitive behavioural therapy on insomnia in adults with tinnitus: Systematic review and meta-analysis of randomised controlled trials. *Sleep Medicine Reviews, 56*, 101405.

Dixon, P. R., Crowson, M., Shipp, D., Smilsky, K., Lin, V. Y., Le, T., et al. (2020). Predicting reduced tinnitus burden after cochlear implantation in adults. *Otology & Neurotology, 41*, 196-201.

Fackrell, K., Hall, D. A., Barry, J. G., Hoare, D. J. (2018). Performance of the Tinnitus Functional Index as a diagnostic instrument in a UK clinical population. *Hearing Research, 358*, 74-85.

Feeny, D., Furlong, W., Boyle, M., Torrance, G. W. (1995). Multi-attribute health status classification systems. Health Utilities Index. *Pharmacoeconomics, 7*, 490-502.

Gagnon, C., Belanger, L., Ivers, H., Morin, C. M. (2013). Validation of the Insomnia Severity Index in primary care. *J Am Board Fam Med, 26*, 701-710.

Gatehouse, S. & Noble, W. (2004). The Speech, Spatial and Qualities of Hearing Scale (SSQ). *International Journal of Audiology, 43*, 85-99.

Genitsaridi, E., Partyka, M., Gallus, S., Lopez-Escamez, J. A., Schecklmann, M., Mielczarek, M., et al. (2019). Standardised profiling for tinnitus research: The European School for Interdisciplinary Tinnitus Research Screening Questionnaire (ESIT-SQ). *Hear Res, 377*, 353-359.

Gomersall, P. A., Baguley, D. M., Carlyon, R. P. (2019). A cross-sectional questionnaire study of tinnitus awareness and impact in a population of adult cochlear implant users. *Ear Hear, 40*, 135-142.

Gopinath, B., McMahon, C. M., Rochtchina, E., Karpa, M. J., Mitchell, P. (2010). Incidence, persistence, and progression of tinnitus symptoms in older adults: The Blue Mountains Hearing Study. *Ear and Hearing, 31*, 407-412.

Hall, D. A., Mohamad, N., Firkins, L., Fenton, M., Stockdale, D. (2013). Identifying and prioritizing unmet research questions for people with tinnitus: The James Lind Alliance Tinnitus Priority Setting Partnership. *Clinical Investigation, 3*, 21-28.

Hall, D. A., Smith, H., Hibbert, A., Colley, V., Haider, H. F., Horobin, A., et al. (2018). The COMiT'ID study: Developing Core Outcome Domains Sets for clinical trials of sound-, psychology-, and pharmacology-based interventions for chronic subjective tinnitus in adults. *Trends in Hearing, 22*, 2331216518814384.

Herdman, M., Gudex, C., Lloyd, A., Janssen, M. F., Kind, P., Parkin, D., et al. (2011). Development and preliminary testing of the new five-level version of EQ-5D (EQ-5D-5L). *Quality of Life Research, 20*, 1727-1736.

Kim, H., Kang, W. S., Park, H. J., Lee, J. Y., Park, J. W., Kim, Y., et al. (2018). Cochlear implantation in postlingually deaf adults is time-sensitive towards positive outcome: Prediction using advanced machine learning techniques. *Scientific Reports, 8*, 18004.

Kloostra, F. J. J., Arnold, R., Hofman, R., Burgerhof, J. G. M., van Dijk, P. (2019). Models to predict positive and negative effects of cochlear implantation on tinnitus. *Laryngoscope Investig Otolaryngol, 4*, 138-142.

Kloostra, F. J. J., Arnold, R., Hofman, R., Van Dijk, P. (2015). Changes in tinnitus after cochlear implantation and its relation with psychological functioning. *Audiology & Neurotology, 20*, 81-89.

Kloostra, F. J. J., Verbist, J., Hofman, R., Free, R. H., Arnold, R., van Dijk, P. (2018). A prospective study of the effect of cochlear implantation on tinnitus. *Audiology and Neurotology, 23*, 356-363.

Knopke, S., Szczepek, A. J., Haussler, S. M., Grabel, S., Olze, H. (2017). Cochlear implantation of bilaterally deafened patients with tinnitus induces sustained decrease of tinnitus-related distress. *Front Neurol, 8*, 158.

Kroenke, K., Spitzer, R. L., Williams, J. B. (2001). The PHQ-9: Validity of a brief depression severity measure. *Journal of General Internal Medicine, 16*, 606-613.

Langguth, B. (2011). A review of tinnitus symptoms beyond ‘ringing in the ears’: A call to action. *Current Medical Research and Opinion, 27*, 1635-1643.

McCormack, A., Edmondson-Jones, M., Somerset, S., Hall, D. A. (2016). A systematic review of the reporting of tinnitus prevalence and severity. *Hearing Research, 337*, 70-79.

Meikle, M. B., Henry, J. A., Griest, S. E., Stewart, B. J., Abrams, H. B., McArdle, R., et al. (2012). The Tinnitus Functional Index: Development of a new clinical measure for chronic, intrusive tinnitus. *Ear and Hearing, 33*, 153-176.

Miguel, G. S., Yaremchuk, K., Roth, T., Peterson, E. (2014). The effect of insomnia on tinnitus. *The Annals of Otology, Rhinology, and Laryngology, 123*, 696-700.

National Institute for Health and Care Excellence. (2009). Cochlear implants for children and adults with severe to profound deafness. In *NICE technology appraisal guidance 166*. Available from: [www.nice.org.uk/guidance/ta166](file:///C:/Users/mszat/AppData/Local/Microsoft/Windows/INetCache/Content.Outlook/4MSP7RER/www.nice.org.uk/guidance/ta166).

National Institute for Health and Care Excellence. (2013). Guide to the methods of technology appraisal. In *NICE technology appraisal guidance 166*. Available from: <https://www.nice.org.uk/process/pmg9/chapter/foreword>.

National Institute for Health and Care Excellence. (2019a). Cochlear implants for children and adults with severe to profound deafness. In *NICE technology appraisal guidance 566*.

National Institute for Health and Care Excellence. (2019b). Position statement on use of the EQ-5D-5L value set for England. In. Available from: <https://www.nice.org.uk/about/what-we-do/our-programmes/nice-guidance/technology-appraisal-guidance/eq-5d-5l>.

National Institute for Health and Care Excellence. (2020). Tinnitus: assessment and management. In *NICE guideline NG155*.

Noble, W., Jensen, N. S., Naylor, G., Bhullar, N., Akeroyd, M. A. (2013). A short form of the Speech, Spatial and Qualities of Hearing scale suitable for clinical use: the SSQ12. *International Journal of Audiology, 52*, 409-412.

Nondahl, D. M., Cruickshanks, K. J., Huang, G. H., Klein, B. E., Klein, R., Nieto, F. J., et al. (2011). Tinnitus and its risk factors in the Beaver Dam offspring study. *International Journal of Audiology, 50*, 313-320.

Olze, H., Szczepek, A. J., Haupt, H., Forster, U., Zirke, N., Grabel, S., et al. (2011). Cochlear implantation has a positive influence on quality of life, tinnitus, and psychological comorbidity. *The Laryngoscope, 121*, 2220-2227.

Olze, H., Szczepek, A. J., Haupt, H., Zirke, N., Graebel, S., Mazurek, B. (2012). The impact of cochlear implantation on tinnitus, stress and quality of life in postlingually deafened patients. *Audiology & Neurotology, 17*, 2-11.

Pan, T., Tyler, R. S., Ji, H., Coelho, C., Gehringer, A. K., Gogel, S. A. (2009). Changes in the tinnitus handicap questionnaire after cochlear implantation. *American Journal of Audiology, 18*, 144-151.

Peter, N., Liyanage, N., Pfiffner, F., Huber, A., Kleinjung, T. (2019). The influence of cochlear implantation on tinnitus in patients with single-sided deafness: A systematic review. *Otolaryngol Head Neck Surg*, 194599819846084.

Pierzycki, R. H., Corner, C., Fielden, C. A., Kitterick, P. T. (2019). Effects of tinnitus on cochlear implant programming. *Trends in Hearing, 23*, 2331216519836624.

Pierzycki, R. H., Edmondson-Jones, M., Dawes, P., Munro, K. J., Moore, D. R., Kitterick, P. T. (2016). Tinnitus and sleep difficulties after cochlear implantation. *Ear and Hearing, 37*, e402-e408.

Pierzycki, R. H. & Kitterick, P. T. (2021). Insomnia, anxiety and depression in adult cochlear implant users with tinnitus. *Ear and Hearing, 42*, 235-243.

Ramakers, G. G. J., van Zanten, G. A., Thomeer, H., Stokroos, R. J., Heymans, M. W., Stegeman, I. (2018). Development and internal validation of a multivariable prediction model for tinnitus recovery following unilateral cochlear implantation: a cross-sectional retrospective study. *BMJ Open, 8*, e021068.

Ramakers, G. G. J., van Zon, A., Stegeman, I., Grolman, W. (2015). The effect of cochlear implantation on tinnitus in patients with bilateral hearing loss: A systematic review. *The Laryngoscope, 125*, 2584-2592.

Singh, G. & Pichora-Fuller, K. M. (2010). Older adults’ performance on the speech, spatial, and qualities of hearing scale (SSQ): Test-retest reliability and a comparison of interview and self-administration methods. *International Journal of Audiology, 49*, 733-740.

Smith, M. T. & Wegener, S. T. (2003). Measures of sleep: The Insomnia Severity Index, Medical Outcomes Study (MOS) Sleep Scale, Pittsburgh Sleep Diary (PSD), and Pittsburgh Sleep Quality Index (PSQI). *Arthritis Rheum, 49*, S184-S196.

Smith, S. S., Kitterick, P. T., Scutt, P., Baguley, D. M., Pierzycki, R. H. (2021). An exploration of psychological symptom-based phenotyping of adult cochlear implant users with and without tinnitus using a machine learning approach. In W. Schlee, B. Langguth, T. Kleinjung, et al. (Eds.), *Progress in Brain Research* (pp. 283-300): Elsevier.

Spitzer, R. L., Kroenke, K., Williams, J. B. W., Löwe, B. (2006). A brief measure for assessing generalized anxiety disorder: The GAD-7. *Archives of Internal Medicine, 166*, 1092-1097.

Summerfield, A. Q., Barton, G. R., Group, U. K. C. I. S. (2019). Sensitivity of EQ-5D-3L, HUI2, HUI3, and SF-6D to changes in speech reception and tinnitus associated with cochlear implantation. *Quality of Life Research, 28*, 1145-1154.

Todt, I., Rademacher, G., Mutze, S., Ramalingam, R., Wolter, S., Mittmann, P., et al. (2015). Relationship between intracochlear electrode position and tinnitus in cochlear implantees. *Acta Oto-Laryngologica, 135*, 781-785.

Tyler, R. S. & Baker, L. J. (1983). Difficulties experienced by tinnitus sufferers. *The Journal of Speech and Hearing Disorders, 48*, 150-154.

UK Cochlear Implant Study Group. (2004). Criteria of candidacy for unilateral cochlear implantation in postlingually deafened adults I: theory and measures of effectiveness. *Ear and Hearing, 25*, 310-335.

Vickers, D., Kitterick, P., Verschuur, C., Leal, C., Jenkinson, L., Vickers, F., et al. (2016). Issues in cochlear implant candidacy. *Cochlear Implants Int, 17 Suppl 1*, 1-2.

Wallhausser-Franke, E., D'Amelio, R., Glauner, A., Delb, W., Servais, J. J., Hormann, K., et al. (2017). Transition from acute to chronic tinnitus: Predictors for the development of chronic distressing tinnitus. *Frontiers in Neurology, 8*, 605.

Wyss, J., Mecklenburg, D. J., Graham, P. L. (2019). Self-assessment of daily hearing function for implant recipients: A comparison of mean total scores for the Speech Spatial Qualities of Hearing Scale (SSQ49) with the SSQ12. *Cochlear Implants Int*, 1-12.

Zeng, F. G., Tang, Q., Dimitrijevic, A., Starr, A., Larky, J., Blevins, N. H. (2011). Tinnitus suppression by low-rate electric stimulation and its electrophysiological mechanisms. *Hear Res, 277*, 61-66.

Zenner, H. P., Delb, W., Kroner-Herwig, B., Jager, B., Peroz, I., Hesse, G., et al. (2017). A multidisciplinary systematic review of the treatment for chronic idiopathic tinnitus. *Eur Arch Otorhinolaryngol, 274*, 2079-2091.
